# Implementation of the open surgery reporting guidelines in ruptured brain arteriovenous malformations: Feasability and adaptations

**DOI:** 10.1016/j.bas.2025.105920

**Published:** 2025-12-29

**Authors:** Lucas Ribeiro, Julien Boetto, Pierre-Henri Lefevre, Kifah Khouri, Marine Le Corre

**Affiliations:** a Department of Neurosurgery, University of Montpellier, Gui de Chauliac Hospital, Montpellier, France; b Institute of Functional Genomics (IGF), Université de Montpellier, CNRS, INSERM, U1191, Montpellier, France; c Department of Neuroradiology, University of Montpellier, Gui de Chauliac Hospital, Montpellier, France

**Keywords:** Ruptured brain arteriovenous malformations, Surgery, Outcome, Prognosis, Guidelines

## Abstract

**Introduction:**

Ruptured brain arteriovenous malformations (rAVMs) are a major cause of hemorrhagic stroke in young adults, often leading to severe neurological morbidity. The recently proposed Open Surgery Reporting Guidelines (OSRG) aim to standardize data reporting in microsurgical AVM studies.

**Research question:**

This study assessed the feasibility of OSRG application in ruptured AVM surgery and identified limitations specific to this acute context.

**Methods and materials:**

A retrospective analysis of 86 patients who underwent microsurgical treatment for rAVMs between 2012 and 2022 was performed. The OSRG, encompassing eight domains and 65 items, was retrospectively applied to each case. Reporting completeness, feasibility challenges, and inter-domain consistency were evaluated. Predictors of poor functional outcome (modified Rankin Scale [mRS] > 2) were determined using uni- and multivariate logistic regression.

**Results:**

The mean age was 45.6 ± 17.2 years, and 59.4 % were female. Complete resection was achieved in 91.6 %, with a mortality rate of 4.6 %. Favorable outcome (mRS ≤2) increased from 53.4 % at 3 months to 72.0 % at last follow-up. WFNS >2 (OR 6.38, 95 % CI 1.50–31.36; p = 0.01) and acute hydrocephalus (OR 6.76, 95 % CI 2.09–25.97; p = 0.01) independently predicted poor outcome. OSRG adherence reached 81.5 % (53/65 items), with full completion in radiological, surgical, and administrative domains, while partial gaps concerned preoperative and adverse events reporting.

**Conclusion:**

Applying the OSRG framework in rAVM surgery is feasible and improves reporting accuracy and transparency. Minor adaptations for emergency settings may further enhance its applicability and facilitate interstudy comparability in vascular neurosurgery.

## Highlights

• **First application of OSRG** to ruptured AVMs, demonstrating **feasibility** and identifying rupture-specific limitations.
• Detailed practical **breakdown of how** OSRG guidelines were integrated in rAVMs.
• Proposed **adaptation of OSRG guidelines** in the specific context of rupture.
• Microsurgery achieves **durable cure and functional recovery in most patients,** WFNS >2 and acute hydrocephalus remaining independent prognostic factors.

## 1 Introduction

Brain arteriovenous malformations (AVMs) are high-flow neurovascular abnormalities that contain low-resistance blood vessels which may rupture and cause devastating cerebral hemorrhage (Chen et al., 2020). While theses malformations were believed to be congenital, recent studies have shown the crucial role played by inflammation in their genesis, growth and rupture (Sugiyama et al., 2022).Although rare (Samaniego et al., 2024), they represent a significant life-long burden particularly in youngest patients that harbor a high risk of fatal bleeding during their life-time (Starke et al., 2013) (Lehecka et al.). Ruptured arterio-venous malformations (rAVM) lead to significant permanent neurological impairment or death in approximately 42 % of patients (Fukuda et al., 2017). AVM treatment strategies may include microsurgery, embolization, radiosurgery or combination of thereof. Recently, the Open Surgery Reporting Guidelines (OSRG) (Ferreira et al., 2024a) were proposed to standardize data reporting and enhance the reproducibility of open microsurgical AVM studies. These guidelines consist of seven domains encompassing perioperative variables. Their systematic implementation aims to reduce heterogeneity between surgical series and facilitate meta-analytic comparison. The primary objective of this study was to assess the feasibility and applicability of the OSRG framework in the specific context of ruptured AVMs, where emergent management and incomplete preoperative assessment may limit data completeness.

## 2 Materials and methods

### 2.1 Study design and participants

We conducted a retrospective review of patients who underwent brain surgery for a rAVM over a ten-year period between January 2012 and December 2022. Inclusion criteria were age ≥15 years with a rAVM and proven diagnosis on pathological findings.

To enhance the rigor and reproducibility of data collection, the Open Surgery Reporting Guidelines (OSRG) were systematically applied (Ferreira, Mitre et al., 2024). These guidelines cover eight domains: (1) Patient Baseline and Clinical Characteristics, (2) AVM Grading and Anatomy (3) Angioarchitecture and Morphology, (4) Definitions and Terminology, (5) Surgeon and Team Experience (6) Surgical Technique and Intraoperative Data, (7) Outcomes and Adverse Events, (8) Funding and Conflict of interests. In our study, these domains were retrospectively adapted to the rupture setting, and completeness of each domain was evaluated.

This study was approved by the national ethics committee (n° IRB00011687). All patients provided informed consent for the retrospective analysis of their clinical data. Data and imaging were analyzed after anonymization in accordance with the Personal Data Protection Act and the code of conduct for responsible use of human tissue and medical research.

### 2.2 Data collection

Data of demographics, clinical and patients’ history (including previous treatment) were obtained from medical record. Radiological status was conducted by senior neuroradiologist and included AVM classification, angioarchitecture and postoperative results. Obliteration was determined on postoperative conventional cerebral angiography (CA). CA was performed immediately postoperatively and at six months, while later follow-up used brain computed tomography angiography (CTA) or magnetic resonance imaging (MRI).

Localization and eloquence were defined according to Lawton and al (Lawton et al., 2010) and Spetzler and al (Spetzler and Martin, 1986). *respectively.* Hematoma volume was measured on 3-mm CT slices using Brainlab Elements software (Brainlab AG, Germany) following the method described by Oulasvirta et al. (2022)

As per OSRG recommendations, each data point was classified by completeness: “fully reported,” “partially reported,” or “unavailable.” Intraoperative data included blood loss, timing, and intraoperative events (domain 4), while adverse events were recorded per the OSRG complication checklist (Ferreira, Cardoso et al., 2024)(Ferreira et al., 2024b). Data completeness was assessed at the end of the collection phase to evaluate the feasibility of OSRG implementation in ruptured AVM cases.

### 2.3 Outcome evaluation and follow-up

The primary outcome was favorable functional status, defined as modified Rankin Scale (mRS) ≤2 at three months. Secondary outcomes included mortality and long-term outcome at last follow-up.

### 2.4 Adverse events

Adverse events were categorized as hemorrhagic, neurologic or ischemic. Each event was documented with details on timing (intraoperative or postoperative), required management, and patient outcomes. Intraoperative bleeding leading to massive transfusion (≥ 10 blood units)(Raymer et al., 2012) and premature rupture of the AVM leading to hemodynamic failure (sudden hemorrhagic shock) were recorded. Overall postoperative adverse events were reported and divided between procedure-related and non-procedure-related complications (e.g., urinary infection, pulmonary embolism). Specific approach and check list were used to improve the data collection of adverse events (Ferreira et al., 2024b). Completeness of adverse event reporting was recorded as part of our OSRG feasibility analysis.

### 2.5 Surgical approach

All surgeries were performed by experienced neurosurgeons (one senior K.K and one early-career neurosurgeon M.L.C.) under general anesthesia. Approaches and patient position were tailored according to the AVM location. Microsurgical resection began with subarachnoid circumferential dissection, identification of arterial feeders, and stepwise isolation of the nidus. The brain hematoma associated with the AVM was removed prior to the occlusion of the draining vein. Indocyanine green angiography (ICG) confirmed complete resection, and neuronavigation was used in all cases. No intraoperative neuromonitoring was applied.

### 2.6 Statistical analysis

Statistical tests were conducted using GraphPad Prism version 10.4.1 (GraphPad Software). Continuous variables were expressed as mean and standard deviation, and qualitative data were described using percentages. Univariable and multivariable logistic regression assessed predictive factors of poor functional outcome and postoperative remnant. Effect measures include odd ratios (OR) with 95 % confidence intervals (CI). Staked bar charts and forests plots were created with GraphPad Prism version 10.4.1. Functional outcome at baseline, 3 months and last follow-up were represented on staked bar charts and multivariable logistic regression for functional outcome on forests plots. Statistical significance was set at p < 0.05.

## 3 Results

### 3.1 Population and follow-up

Eighty-six patients (51 female, 59.4 %) were treated by microsurgery for a rAVM. Mean age was 45.6 ± 17.2 years (range 15–78). Mean follow-up was 46.2± 38.3months (range 6–144). Baseline data and history (i.e,WFNS score, seizure, n° of ruptures, previous treatment) are detailed in Table 1.

**Table 1:** Baseline data summary of rAVM treated by microsurgery (n = 86).

Characteristic	Value∗
Mean age (years) ± SD	45.6 ± 17.2
Sex (%male)	35 (40.6)
Associated bAVM syndromes (%)	0 (0.0)
History of seizures (%) Two seizures or more	12 (13.9) 10 (83.3)
**Baseline WFNS score** (%)	
≤ 2.	32 (37.2)
> 2	54 (62.8)

**Glasgow Coma Scale** (%)	
15	35 (40.6)
14−8	28 (32.6)
<7	23 (26.8)

**Second episode of rupture (%)**	6 (7.0)

**Location** (%)	
Frontal	21 (24.5)
Parieto-occipital	27 (31.3)
Temporal	17 (19.7)
Deep	4 (4.7)
Ventricular	2 (2.3)
Cerebellar	15 (17.5)

**Spetzler-Martin Grade**	
I	19 (22.0)
II	32 (37.2)
III	23 (26.8)
IV	12 (14.0)

**Angioarchitecture**	
Proximal feeding artery (%)	
sylvian artery	56 (65.1)
posterior cerebral artery	20 (23.2)
anterior cerebral artery	16 (18.6)
two or more arteries involved	29 (33.7)
Superficial drainage (%)	47 (54.7)
Venous ectasia or outlet (%)	17 (19.7)
Intranidal aneurysm (%)	2 (2.3)
Intranidal fistulae (%)	1 (1.2)
Associated Proximal Brain Aneurysm (%)	12 (13.9)

### 3.2 AVM characteristics

Mean hematoma volume was 31.4 ± 22.6 cc (range 2–89). Intraventricular hemorrhage was present in 46 (53.4 %), and acute hydrocephalus in 37 (43.0 %) with external ventricular drainage performed in 27 (31.3 %). Spetzler-Martin (SM) grade I-II were most common (n = 51, 59.3 %). The supplementary grade (Kim et al. 2015) was ≤6 in 77 (89.5 %). AVMs were left-sided in 41 (52.5 %) and involved eloquent regions in 43 (50.0 %). All nidus were compact, mean nidus size was 2.3 ± 1.1 cm (range 0.5–5), maximal nidus diameter was < 3 cm in 61 (70.9 %), between 3 and 6 cm in 25 (29.1 %) and none had diameter over 6 cm. Overall angioarchitecture's features are detailed in Table 1.

### 3.3 Surgical details

Microsurgery was the primary treatment in 78 cases (90.6 %), with preoperative embolization in 6 (7.0 %) and hematoma evacuation alone in 2 (2.4 %). Eleven patients (12.7 %) underwent preliminary external ventricular drainage or decompression. AVM resection was performed within the first 7 days after rupture (early surgery) in 72 patients (83.7 %), and after 7 days (delayed surgery) in 14 patients (16.3 %). Mean length of the procedure was 195.7 ± 45.3min (range 77.2min–320.4min) with a mean blood loss during AVM resection of 713.6± 833.7 cc (range 150–4000 cc).

### 3.4 Functional outcome

**Fig. 1:**
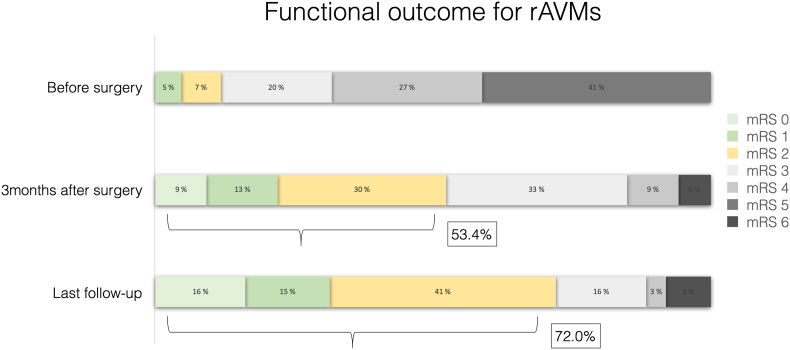
Functional outcome of 86 rAVM cases treated with surgery.

Favorable outcome (mRS≤2) at three months was achieved in 46 (53.4 %) and at last follow-up in 62 (72.0 %) (Fig. 1). Improvement ≥1 mRS point was noted in 70/86 (81.3 %) patients at 3months. Predictors of poor outcome at 3 months and final follow-up were acute hydrocephalus, WFNS >2, and hematoma >30 cc; only acute hydrocephalus and WFNS >2 remained significant on multivariate analysis (Table 2). New onset of seizures in previously non-epileptic patients was recorded in 8/74 (10.8 %) ranging from 1 month to five years postoperative with 50.0 % occurring after 2 years (4 of 8). Complete relief of seizures in previously epileptic patients occurred in 2/12 (16.7), rare seizure controlled under treatment in 9/12 (75.0) and occasional seizure controlled under treatment in 1/12 (8.3).

**Table 2:** Univariate and multivariate logistic regression for poor functional outcome (mRS >2).

Variable	Univariate OR (95 % CI)	p-value	Multivariate OR (95 % CI)	p-value
• WFNS >2	4.09 (2.30–8.48)	<**0.001**	6.38 (1.50–31.36)	**0.01**
• Hematoma volume >30 cc	2.12 (1.43–3.45)	<**0.001**	1.62 (0.44–6.05)	**0.48**
• Acute hydrocephalus	1.89 (1.31–2.95)	<**0.001**	6.76 (2.09–25.97)	**0.01**
• Female sex	1.05 (0.77–1.44)	0.74	–	–
• Age >45 years	1.88 (0.49–7.68)	0.35	–	–
• SM grade > II	2.86 (0.59–15.32)	0.19	–	–
• Eloquent location	1.15 (0.84–1.61)	0.37	–	–
• Deep venous drainage	1.57 (0.67–3.74)	0.29	–	–

Abbreviations: OR = odds ratio; CI = confidence interval; WFNS = World Federation of Neurosurgical Societies; SM = Spetzler–Martin grade. Statistically significant values (p < 0.05) are highlighted in bold.

### 3.5 Radiological outcome

Postoperative complete resection was achieved in 77/84 (91.6 %). Remnant AVM was observed in 7 (8.4 %), and no recurrences were seen at follow-up. Retreatment achieved full obliteration in five of these cases, yielding a final rate of 95.3 %. No association was found between risk of remnant and AVM grading, age, acute hydrocephalus, hematoma volume and WFNS status.

### 3.6 Adverse events and mortality

Intraoperative premature rupture occurred in one case, and no massive transfusion was required. Four patients (4.6 %) died postoperatively due to AVM rupture; three additional deaths were unrelated. One patient had postoperative epidural hematoma requiring revision surgery, six ischemic strokes (7.0 %) occurred, two causing permanent deficits and infection occurred in five (5.8 %). Two had brain swelling needing decompressive craniectomy. Overall procedure-related complications were 16.3 % (n = 14). Overall non-related procedure complications occurred in 9 patients (10.4 %).

### 3.7 OSRG feasibility and reporting challenges

Overall adherence to the OSRG reached 81.5 % (53/65 items). Data completeness per domain was as follows: Domain 1 – Baseline characteristics, 82 %; Domain 2 – Grading and radiological aspects, 100 %; Domain 3 – Angioarchitecture, 75 %; Domain 4 – Pivotal concept definitions, 50 %; Domain 5 – Neurosurgeon and staff characteristics, 100 %; Domain 6 – Surgical details, 100 %; Domain 7 – Clinical and surgical outcomes, 75 %; and Domain 8 – Funding and conflicts of interest, 100 %. Completeness was highest in domains describing radiological grading, surgical details, and administrative transparency, whereas partial reporting mainly affected pivotal concept definitions (complications and adverse events classification) (Table 3).

**Table 3:** Details of unreported or partially reported OSRG items and reasons.

OSRG Domain	N° Items	Item(s) Unreported or Partially Reported	Reason for Incompleteness	Impact/Comment
**1. Baseline characteristics**	11	1c, 1g	Comorbidities and long-term follow-up not consistently recorded in retrospective files.	May affect outcome interpretation in the long run.
**3.Angioarchitecture assessment**	8	3d, 3e	Venous stenosis, intranidal fistula not available in older imaging.	Reflects emergency and ruptured-specific AVM distortion
**4. Pivotal concept definitions**	8	4c, 4d, 4e, 4g	No validated perioperative event scale.	Reduces comparability; future studies should adopt standardized definitions.
**7. Outcomes and adverse events**	16	7f, 7g, 7j, 7m	No validated adverse event classification or subgroup analysis performed.	Hinders interstudy comparison.

## 4 Discussion

### 4.1 Implementation of the open surgery reporting guidelines

This study represents, to our knowledge, the first systematic attempt to apply the OSRG to ruptured AVMs. The Open Surgery Reporting Guidelines, introduced by Ferreira et al. (2024), include eight domains encompassing 65 items that serve as a structured checklist to improve transparency, reproducibility, and interstudy comparability in research on open surgery for brain arteriovenous malformations.

Implementation of the OSRG in this retrospective series of rAVMs demonstrated that the framework is feasible and informative for improving data quality, consistency, and interstudy comparability. Of the 65 checklist items distributed across eight domains, 53 (81.5 %) were completely reported, 12 (18.5 %) partially reported, and none entirely unavailable.

Missing data mainly concerned preoperative details, reflecting the inherent limitations of retrospective emergency cases, but also underscored the need for clear pivotal concept definitions in vascular neurosurgery, particularly for adverse events, to ensure comparability with other studies. Despite these limitations, all critical domains—including radiological grading, surgical details and outcome—were fully addressed. These findings underscore that even in the context of ruptured AVMs, the OSRG can be successfully applied with minimal adaptation, providing a reproducible framework for comprehensive data reporting and future multicenter comparability (Table 4).

**Table 4:** Implementation of the open surgery reporting guidelines (OSRG) in ruptured AVMs.

OSRG Domain	Purpose	How It Was Applied in the Present Study	Limitations in Ruptured AVMs	Potential Impact
**1. Patient Baseline and Clinical Characteristics**	Ensure comparability across series and capture preoperative condition.	WFNS score, GCS, age, sex, seizure history, previous-AVM bleeding systematically recorded.	Lack of validated rAVM-specific grading systems for clinical status	Improved baseline data completeness
**2. AVM Grading and Anatomy**	Risk stratification and reproducibility.	Spetzler–Martin and Supplementary grades reported for all; lesion side and eloquence specified.	Hemorrhage-induced distortion limited grading accuracy in certain cases	Revealed need to record whether grading was done pre- or post-rupture.
**3. Angioarchitecture and Morphology**	Describe feeding arteries, drainage, and nidus configuration for surgical planning.	Arterial supply, venous drainage, intranidal aneurysm, and nidus compactness collected from CA.	Vessel collapse or thrombosis post-rupture hindered full evaluation in emergency angiograms.	Improved anatomical documentation; established feasibility of OSRG angiographic assessment even in acute setting. Revealed potential need for delayed re-apparaisal of angiography when feasible.
**4. Definitions and Terminology**	Standardize reporting of hemorrhage, obliteration, and complications.	Adopted common terminology for obliteration (‘complete’ vs ‘residual’) and outcome (mRS ≤2).	Lack of validated peri-operative event definitions.	Exhibited the need for standardized reporting definitions in vascular neurosurgery
**5. Surgeon and Team Experience**	Acknowledge learning curve and institutional expertise.	All surgeries performed by two senior vascular neurosurgeons at one center.	__	Conferred internal consistency and transparency for reproducibility.
**6. Surgical Technique and Intraoperative Data**	Support reproducibility of procedures	Approach, patient position, hematoma evacuation, timing (early vs delayed), and use of ICG recorded.	Small Deviations from Standard Protocol in Emergency Settings	Highlighted the role of standardized surgical protocol
**7. Outcomes and Adverse Events**	Standardize outcome measures and complications.	Functional outcome (mRS 3 mo, final), angiographic cure, mortality, and new-onset epilepsy recorded. Adverse events divided into rupture-related vs. procedure-related.	Attribution bias—rupture severity overlaps with surgical risk.	Clearer identification of independent prognostic factors (WFNS >2, hydrocephalus). Framework improved transparency of morbidity assessment.

### 4.2 Adaptation of the open surgery reporting guidelines in rAVMs

Despite OSRG feasibility, rupture settings might introduce distinctive barriers in outcome interpretation, bAVMs morphological assessment or intraoperative documentation. Hematoma-related mass effect or vessel thrombosis distort anatomical grading (Domain 2–3) and preclude precise AVM grading. In emergency surgery, documentation of intraoperative variables (Domain 4–6) may be incomplete due to deviation from standard protocol, and outcomes (Domain 7) are frequently confounded by rupture severity rather than treatment effect. To overcome these limitations, we propose a series of targeted, rupture-specific adaptations to the OSRG framework. (Table 5).

**Table 5:** Proposed rupture-specific OSRG extensions.

Proposed Variable → Domain Link	Rationale	Impact on Reporting Quality
**Time from rupture to surgery (early versus delayed)** Question: “Is the interval between rupture and surgery clearly reported, with definitions of “early” and “delayed” provided?” → **Domain 6**	Discrepancies in the literature as whether the surgical timing as an impact on functional outcome. Timing cut off to be defined.	Improve outcome evaluation.
**Deferred grading/delayed DSA reassessment** Question: “Did the authors report the use of deferred grading and document any delayed DSA reassessment following stabilization or hematoma evacuation/resolution?” → **Domain 2**	Hemorrhage often prevents accurate grading.	Enables consistent grading.
**Anatomical distortion (thrombosis, vessel collapse, mass effect)** Question: “Did the authors report the presence and type of anatomical distortion (thrombosis, vessel collapse, mass effect) and indicate whether it hindered accurate AVM characterization in the acute setting?” → **Domain 3**	Common in rupture, affects angioarchitecture interpretation.	Improves validity of morphological comparisons.
**Protocol deviations for emergencies** Question: “Did the authors report any protocol deviations required by the emergency situation and clarify how these deviations affected data completeness?” → **Domain 6**	Sometimes necessary in urgent cases.	Ensures transparency and preserves reproducibility.
**Differentiation of rupture- vs. procedure-related complications** Question: “Did the authors differentiate rupture-related complications from procedure-related complications and report them separately?” → **Domain 7**	Key for fair assessment of surgical risk.	Prevents overestimation of surgical morbidity.
**Resource-use metrics (length of stay, blood-loss volume, procedure duration)** Question: “Did the authors report resource-use metrics (length of stay, procedure duration, blood-loss volume) as part of the peri-operative description?” → **Domain 1/6**	Complements outcome evaluation with technical complexity and severity of rupture.	Captures case complexity, perioperative burden and enhance comparability across centers.

### 4.3 Microsurgical outcomes

Microsurgery remains the most definitive curative option for rAVMs providing durable protection from bleeding with high cure rates (Korja et al., 2014) (Aboukaïs et al., 2015) (Potts et al., 2015). Here, complete excision was achieved in 91.6 % with a procedure-related morbidity of 16.3 % and mortality of 4.6 %, values that are consistent with contemporary reports (Korja et al., 2014) (Potts et al., 2015). The high obliteration rate underscores the efficacy of microsurgery in experienced hands, particularly for Spetzler–Martin (SM) grades I–III and supplementary scores ≤6 (Lawton et al., 2010). Outcome quality is highly dependent on the surgeon's understanding of AVM angioarchitecture. Although we did not observe a statistical correlation between SM grade and residual nidus, this likely reflects the limited sample size and the predominance of low-grade lesions. High-grade or eloquent AVMs remain technically challenging and are often best managed through a staged or multimodal approach combining surgery, embolization, or radiosurgery.

### 4.4 Functional recovery and predictors of outcome

Favorable outcome (mRS ≤2) increased from 53.4 % at three months to 72.0 % at last follow-up, with measurable clinical improvement in over 80 % of patients. This confirms that many patients continue to recover neurologically long after the acute phase, a finding also emphasized by Lawton et al. (2005) (Lawton et al., 2005) and Murthy et al. (2017) (Murthy et al., 2017).

Univariate analysis identified baseline WFNS >2, acute hydrocephalus, and hematoma volume >30 cc as significant predictors of poor prognosis; however, only WFNS >2 and acute hydrocephalus remained independent predictors on multivariate analysis (p < 0.05), consistent with Aboukaïs et al. (2015) (Aboukaïs et al., 2015) and Maalim et al. (2023) (Maalim et al., 2023). These results underscore the critical influence of initial clinical severity and ventricular obstruction on postoperative recovery.

The parenchymal injury caused by the initial hemorrhage is the main source of neurological impairment, with long-lasting devastating effects on brain function regardless of treatment decision or AVM grading (Aboukaïs et al., 2017a). Moreover, the negative impact increases when hemorrhage spreads into surrounding healthy white matter bundles (Jiang et al., 2019). The volume equally influences outcome, as larger hematomas were associated with poorer prognosis (Oulasvirta et al., 2022).

Also, hematomas in the posterior fossa differ from supratentorial locations, as patients deteriorate with smaller volumes due to brainstem compression or hydrocephalus (Rodríguez-Hernández et al., 2012) (Magro et al., 2020). On these observations and the literature, we advocate early hematoma evacuation as soon as neurological deterioration occurs rather than deferring until volume exceeds 30 cc. For low-grade lesions, AVM excision may be performed during the same procedure; for higher-grade AVMs, staged resection after stabilization remains prudent (Beecher et al., 2018).

### 4.5 Follow-up & de-novo epilepsy

Clinical long-term follow-up is extremely important to appropriately evaluate the functional outcome as many patients will improve over time as demonstrated here. Prolonged radiological surveillance is essential in rAVM patients. Early postoperative angiography identifies most residual nidus, for which prompt retreatment is recommended to prevent rebleeding. True recurrence is rare and typically delayed, predominantly affecting younger patients with deep venous drainage (Aboukaïs et al., 2017b) (Järvelin et al., 2023). Subsequent follow-up with MRI is sufficient for long-term verification of occlusion. Extended follow-up revealed detection of seizures in 10.8 % of previously non-epileptic patients, occurring between one month and five years postoperatively, with half arising after two years. This topic was poorly documented until recently (Sen et al., 2022), where the one-year cumulative rates of developing *de novo* epilepsy was found between 8.5 and 13.8 % (Sen et al., 2022) (Sioutas et al., 2023). Sen and al (Sen et al., 2022)showed that mean-time for developing *de novo* seizure was about 26.0 months with longest onset being 14 years after surgical treatment. Temporal-lobe AVMs presenting with hemorrhage appear particularly prone to poorly controlled seizures. Finally, and from our perspective, a two-year minimum clinical follow-up seems appropriate to properly conclude on patient functional outcome, whereas five years or more is advisable to capture the majority of delayed seizures and possible recurrences.

## 5 Limitations

The retrospective monocentric nature of our study was the main limitation. Our small sample size hindered our ability to show possible associations on regression analysis. Patients excluded from surgery were not included neither studied and therefore this study does not allow us to draw unbiased conclusions about the estimated surgical risk for either low- and high-grade AVMs. Long-term follow-up is heterogeneous among patient's, possibly hindering the real estimation of favorable outcome in the long run.

## 6 Conclusions

This eleven-year experience demonstrates that implementation of the Open Surgery Reporting Guidelines (OSRG) in ruptured brain arteriovenous malformations is both feasible and informative. The framework significantly improved data completeness, consistency, and transparency, even within the constraints of emergent surgery and retrospective data collection. While certain items were partially limited by emergency, overall adherence exceeded 80 %. Applying the OSRG facilitated clearer identification of prognostic factors and strengthened our reporting accuracy. These findings support the OSRG as a robust tool for standardizing surgical reporting even in acute setting. Prospective adoption of the guideline could enhance comparability across centers, promote multicenter data integration, and ultimately improve the quality of evidence in AVM open surgery research.

## Disclosures

The authors have no personal, financial, or institutional interest in any of the drugs, materials, or devices described in this article.

## Previous presentation

None.

## Previous publication

None.

## Funding

None.

## Declaration of competing interest

The authors declare the following financial interests/personal relationships which may be considered as potential competing interests:Lucas RIBEIRO reports article publishing charges was provided by Hospital Gui de Chauliac Department of Neurosurgery. If there are other authors, they declare that they have no known competing financial interests or personal relationships that could have appeared to influence the work reported in this paper.
